# Folic Acid-Functionalized Albumin/Graphene Oxide Nanocomposite to Simultaneously Deliver Curcumin and 5-Fluorouracil into Human Colorectal Cancer Cells: An *In Vitro* Study

**DOI:** 10.1155/2023/8334102

**Published:** 2023-05-31

**Authors:** Hassan Bardania, Farajollah Jafari, Marzieh Baneshi, Reza Mahmoudi, Maryam Tajali Ardakani, Farshad Safari, Mehrzad Jafari Barmak

**Affiliations:** ^1^Cellular and Molecular Research Center, Yasuj University of Medical Sciences, Yasuj, Iran; ^2^Student Research Committee, Yasuj University of Medical Sciences, Yasuj, Iran; ^3^Department of Chemistry, Cape Breton University, 1250 Grand Lake Road, Sydney, Nova Scotia, Canada B1P 6L2

## Abstract

**Background:**

Nowadays, due to various inherent properties, graphene-based nanoparticles are widely used in drug delivery research. On the other hand, folate receptors are highly expressed on the surface of human tumor cells. In this work, to enhance the 5-fluorouracil (5FU) and curcumin (Cur) effects on colon cancer, we constructed a folic acid- (FA-) modified codelivery carrier based on graphene nanoparticles (GO-Alb-Cur-FA-5FU).

**Materials and Methods:**

The HUVEC and HT-29 were selected for evaluating the antitumor effect of the prepared nanocarriers. The structure of nanocarriers was characterized by FTIR spectroscopy, X-ray diffraction analysis, TEM microscopy, and a DLS analyzer. The efficiency of the prepared carrier was evaluated by fluorescence microscopy using Annexin V and the PI kit. The cytotoxicity of the carrier's component individually and the efficacy of the drug carrier GO-Alb-Cur-FA-5FU were assessed by MTT.

**Results:**

The results of the pharmacological tests indicated that the new nanoparticles cause increased apparent toxicity in HT-29 cells. The apoptosis rate of the HT-29 and HUVEC cells treated with IC50 values of GO-Alb-Cur-FA-5FU for 48 h was higher than the cells treated with IC50 values of 5FU and Cur individually, which indicated the greater inhibitory efficacy of GO-Alb-Cur-FA-5FU than free drugs.

**Conclusion:**

The designed GO-Alb-CUR-FA-5FU delivery system can be applied for targeting colon cancer cells and can be severe as a potential candidate for future drug development.

## 1. Introduction

Colon cancer, known as one of the most devastating diseases, causes cell disorders, genetic changes, and a significant mortality rate subsequently [1, 2]. Based on the statics, colorectal cancer is the third leading cause of cancer-related death in the United States [3, 4]. Colorectal cancer starts in the innermost layer (the mucosa) and can grow outward through some or all of the other layers [5, 6]. When cancer cells proliferate in the wall, they can attack blood and lymphatic vessel lymph nodes, or distant parts of the body [7–9]. Various agents could be used to treat this disease including 5-fluorouracil (5FU) and curcumin (Cur) [10–12]. As a cytotoxic antimetabolic drug and also a chemotherapy agent, 5FU competes with the thymidine synthesis enzyme. The use of this conventional and chemical anticancer agent has been limited due to these drugs effects on cells nonspecifically. To overcome this barrier, a targeted drug delivery system which includes the drug, carrier, and target linker has been used. The biological behavior of the carrier and its linker determines its absorption, distribution, and metabolization. Therefore, a carefully designed and constructed target vector and linker deliver a specific dosage of a drug to the target cells [13].

Graphene oxide (GO), the oxidized form of graphite, a two-dimensional carbon material, contains several hydrophilic functional groups on the basal plane and its edges [14, 15]. The abundant surface area and presence of *π* electrons on the GO surface enable *π*-*π* interactions, leading to a high loading capacity of the drug which along with its acceptable biocompatibility have sparked a lot of interest in different biomedical fields such as drug delivery, bioimaging, cancer therapy, tissue engineering, and wound healing [16, 17]. GO and GO-based nanocomposites showed the potential to be effective nanocarriers for the delivery of different anticancer drugs such as sumatriptan succinate, methotrexate, quercetin, 5FU, cisplatin, and Cur [18, 19]. Recent developments have considered the use of a polysaccharide bionanocomplex along with GO in pharmaceutical applications, such as GO, chitosan, cellulose, albumin, and gelatin to enhance its stability, biocompatibility, solubility in water and physiological medium, and also its dose-dependent cytotoxicity [16, 20–23].

5FU, an antimetabolite drug, has been attributed to incorporating fluoronucleotides in place of nucleotides that inhibit the nucleic acid synthetic enzyme thymidylate synthase (TS), hence exhibiting cytotoxicity [20]. It has been used in the treatment of a range of cancers such as colorectal, breast, and gastrointestinal. Although dihydropyrimidine dehydrogenase (DPD), an enzyme abundantly expressed in the liver, catabolizes more than 80% of administered 5FU to dihydrofuorouracil (DHFU), 5FU upregulates several survival signals including NF-*κ*B and Akt. The NF-*κ*B pathway is a major effect or pathway that leads to chemoresistance [24]. Despite all these, the toxicity and limited solubility of 5FU are barriers to its applications. GO-based materials have been explored for 5FU with the goal of improving the therapeutic efficiency as well as reducing its toxicity. Besides that, GO can enhance the solubility, stability, and control the release behavior of the 5FU resulting in its side effects.

Cur, a natural polyphenol from the root of Curcuma longa Linn, shows anticancer potential despite its hydrophobicity and basic pH [12, 25]. Different mechanisms have been reported for Cur toward cancer cells including mitogen-activated protein kinases (MAPK), NF-*κ*B, activator protein-1, IkBa kinase, 5-lipoxygenases, cyclooxygenase-2, urinary plasminogen activator, tumor necrosis factor, inducible nitric oxide synthase, chemokines, and cell cycle machinery [8]. Since it can downregulate NF-*κ*B, both directly and through the TS pathway, it can be used to circumvent drug resistance. Despite its advantages, its poor solubility and rapid metabolism limit its therapeutic efficacy, and some nanoparticles were used in previous studies for improving its solubility [26, 27]. Recently, the use of GO-based materials as a drug delivery system for Cur has been explored as a means of overcoming these limitations. This system was not only found to enhance the solubility and stability of Cur but also improve its bioavailability and target specificity. In addition, GO can form stable nanocomposites with Cur which protect it from rapid degradation, allowing it to reach the target site more efficiently.

Folic acid (FA) is vital for the maintenance and proliferation of all cells, and its receptors are overexpressed on the surface of many human tumor cells, such as ovarian, lung, breast, endometrial, kidney, and colon cancers [28]. The significant upregulation of folate receptors in tumor tissues has led to the hypothesis that folic acid-related therapeutic agents may exhibit reduced onsite toxicity and enhanced potency against tumor cells compared to nontargeted drugs. FA and FA conjugates can bind to the folate receptors (FRs) with high affinity by receptor-mediated endocytosis, and the FA-modified drug delivery vectors can transfer the therapeutic agents to tumor cells that exhibit amplified folate receptor expression subsequently [29, 30].

With the aim of having a new formulation drug delivery system with higher biostability, lower toxicity, higher tumor-targeting ability, and controlled drug release for efficient cancer treatments, here for the first time, the synergic delivery of 5FU and Cur by a folic-acid-decorated human serum albumin-coated GO nanocarrier (GO-ALB-FA) nanocarrier against folate-receptor overexpressing HT-29 cancer cells [31] has been evaluated. While the proposed system enhances the solubility and bioavailability of the loaded agents, the presence of Cur can reduce the chemoresistance of cells toward 5FU.

## 2. Materials and Methods

### 2.1. Materials

Graphite powder, sulphuric acid (H2SO4), hydrochloric acid (HCl), sodium nitrate (NaNO3), potassium permanganate (KMnO4), 1-ethyl-3-[3-(dimethylamino) propyl] carbodiimide hydrochloride (EDC), 5-Fluorouracil (5FU), 1-(4,5-dimethylthiazol-2-yl)-3,5-diphenylformazan (MTT), Annexin V, and propidium iodide (PI) were purchased from Sigma-Aldrich.

Human colorectal cancer (HT29) cell lines were provided by the Pasture Institute (Tehran, Iran) and were cultured in RMPI1640 medium supplemented with 10% FBS (Sigma-Aldrich), 2 mM glutamine (Gibco-Life Technologies), and 1% penicillin/streptomycin (Invitrogen, Life Technologies). The Cur drug was provided by the Exir Nano Sina Co. (Tehran, Iran). Folic acid was purchased from Biobasic, Canada, Inc.

### 2.2. Preparation of Graphene Oxide (GO)

GO was prepared through a modified Hummer's method as follows [32, 33]: 0.5 mg of graphite powder was dispersed into a mixture of 25 mL of concentrated sulfuric acid and 0.5 mg of sodium nitrate placed in an ice bath followed by the addition of 3 g of potassium permanganate. The reaction mixture was then stirred at 35°C. After 30 min, the reaction was terminated by adding 100 mL of hot distilled water, followed by adding 10 ml of hydrogen peroxide (30%) and stirring at room temperature. Finally, the suspension was centrifuged at 1000 rpm for 10 min and washed 3 times with 0.1 M hydrochloric acid and distilled water to the neutral pH. The sediment was collected and dried at 50°C in an oven for 3 days. Finally, 10 mg of an aqueous suspension of GO was sonicated using an ultrasonic probe at 60% amplitude (on 4 s/off 2 s) for 1 hr in an ice bath. The aqueous suspension was then centrifuged at 12000 rpm for 10 min to remove the GO bulk sedimented. The GO solutions were then lyophilized and stored in vials for further use.

### 2.3. Preparation of Cur Loaded GO

For loading Cur into GO, 0.5 mg of Cur was dissolved in 2 mL of ethanol and added to 2 mL (2 mg.mL^−1^) of the GO suspension. The mixture was stirred for 6 hrs at 4°C. To separate the unbound Cur from GO, the mixture was transferred to a dialysis bag and immersed in 1 L of PBS containing 5% ethanol for 24 hrs. The final solution was collected and stored at -4°C for further use.

### 2.4. Preparation of GO-Alb-5FU Nanocomposite

4 mg of albumin, 1 mg of 5FU, and 5 mg of EDC were dissolved into 10 mL of distilled water and stirred for 30 min, followed by the addition of 2 mL (2 mg.mL^−1^) of GO suspension. The mixture was stirred on a stirrer for 4 hrs at 4°C. To separate unbound 5FU, the final mixture was then transferred to a dialysis tube and purified against distilled water for 24 hrs. The final product was collected and stored at -4°C for further use.

### 2.5. Preparation of GO–Alb-5FU-Cur Nanocomposite

0.5 mg of Cur was dissolved in 2 mL of ethanol and was added to 2 mL (2 mg.mL^−1^) of GO suspension and stirred for 4 hrs at 4°C. Then, 4 mg of albumin, 1 mg of 5FU, and 5 mg of EDC were added to the above mixture and stirred for 4 hrs at 4°C. The solution was then transferred into a dialysis tube to separate unbound drugs and purified against distilled water for 24 hrs.

### 2.6. FA Modification of GO–Alb-5FU-Cur Nanocomposite

0.5 mg of Cur dissolved in 2 mL of ethanol was added to 2 mL (2 mg.mL^−1^) of GO suspension and stirred for 4 hrs at 4°C. Then, 4 mg of albumin, 1 mg of 5FU, and 5 mg of EDC were added to the above mixture and stirred for 30 min at 4°C. Then, 2 mg of folic acid and 5 mg of EDC were added and stirred for 4 hrs at 4°C. The solution was then poured into dialysis bags, and the dialysis bag was placed in an Erlenmeyer flask containing one liter of distilled water for 24 hrs, and then the solution was transferred to the falcon. To evaluate the conjugation of FA to GO nanocomposite, the final mixture was centrifuged, and their supernatant was used to examine the amount of free FA. The amount of FA loaded was calculated by using

(1) $$\begin{array}{c} \text{The amount of FA loaded}\left(\%\right)=\frac{W_{D0}–W_{D1}}{W_{\text{total}}\times 100}, \end{array}$$

where *W*_*D*0_ and *W*_*D*1_ are initial weight of FA and the weight of unbound FA, respectively, and *W*_total_ represents the total weight of nanoparticles.

The concentration of FA in supernatants was evaluated by a Kanavar HPLC system equipped with a UV-VIS 2550 detector and a Zorbax SB-C18 column (250 mm/3.9 mm, particle size 5 *μ*m). The injection volume was 20 *μ*L, and the mobile phase was a mixture of 60% methanol, 40% water with 1% phosphoric acid at a flow rate of 1 mL.min^−1^. The detector wavelength was set at 280 nm.

### 2.7. Evaluation the Amount of 5FU and Cur Loaded in Nanoparticles

After the drugs were loaded onto graphene nanoparticles, the samples were centrifuged (10 min with 10000 rpm), and their supernatant was analyzed to assess the amount of free drug. The amount of drugs loaded was calculated by using

(2) $$\begin{array}{c} \text{The amount of drugs loaded}\left(\%\right)=\frac{W_{D0}–W_{D1}}{W_{\text{total}}\times 100}, \end{array}$$

where *W*_*D*0_ and W_D1_ are initial weight of drug and the weight of unloaded (free) drug, respectively. Moreover, *W*_total_ represents the total weight of nanoparticles.

The concentration of Cur and 5FU drugs in supernatants was assessed by a Kanavar HPLC system, equipped with a UV-VIS 2550 detector and a Zorbax SB-C18 column (250 mm/3.9 mm, particle size 5 *μ*m). The injection volume was 20 *μ*L, and the mobile phase was a mixture of 60% methanol: 40% water with 1% phosphoric acid and 30% acetonitrile: 70% water with 1% acetic acid for 5FU and Cur, respectively, at a flow rate of 1 mL.min^−1^. The detector wavelength was set at 420 nm and 260 nm for Cur and 5Fu, respectively.

### 2.8. Drug Release

10 mL of the prepared nanocarrier suspension was transferred to a dialysis bag and was immersed in a beaker containing 20 mL of PBS placed on a shaker at 150 rpm and 37°C. At the interval times of 1, 2, 3, 6, 12, 24, 48, and 72 hrs, the releasing media was replaced with fresh PBS and was analyzed with HPLC to detect the Cur and 5FU concentration.

### 2.9. Characterization

X-ray diffraction (XRD) analysis was used to characterize the crystalline nature and phase purity of the assynthesized GO. This analysis was done using an X' Pert Pro system having Cu K radiation with *λ* = 1.54060 Å, at 30 mA and 45 kV at the 2*θ* = 2–70° and scanning rate of 2°/min to show the significant changes in GO crystallization at each oxidation stage [32]. The morphology of the GO-Alb-Cur-5FU-FA nanocomposite was evaluated by a transmission electron microscope (TEM) (JEM-1010; JEOL, Tokyo, Japan). The analysis of the functional group was done using a Shimadzu Corporation FTIR instrument (2808, Japan).

### 2.10. *In Vitro* Antitumor Study

The HUVEC and HT-29 were selected for evaluation of the antitumor effect of the prepared nanocarrier using an MTT assay. After cell subculture into a 96-well plate at a density of 1 × 10^4^ cells per well, the plates were cultivated in an incubator with 5% CO_2_ at 37°C for 24 h. Then, the wells were treated in the following groups at different concentrations: GO, GO-Cur, GO-5FU, GO-Alb-Cur-5FU, GO-Alb-Cur-5FU-FA, free Cur, and free 5FU. After 48 h, the supernatants of the wells were removed, and 20 *μ*L of MTT solution was added to each, followed by incubation at 37°C for 4 h. Then, the supernatants were replaced with 150 *μ*L of dimethyl sulfoxide and were mixed on a shaker at 130 rpm for 15 min to dissolve the formed formazan crystals. The absorption of the 96-well plate was measured at 570 nm using a microplate reader and used to calculate the inhibition rate.

### 2.11. Apoptosis Study

Apoptotic cells were quantified by an Annexin V and PI (propidium iodide) costaining assay. Briefly, after the treatment and at the end of the 24 h incubation period, the HUVEC and HT-29 cells were centrifuged at 1800 rpm for 5 min, and 5 *μ*L of Annexin V and 5 *μ*L of PI were added to each tube. The concentration of Cur and 5FU in free form or encapsulated into nanoparticles were 32 and 200 *μ*g/mL, respectively. The concentration of nanoparticles without drugs was equivalent to nanoparticles containing drugs. The tubes were incubated for 15 minutes in the dark followed by analysis by flow cytometer (Becton Dickinson FACS Calibur flow cytometer; Annexin V-FITC binding was detected using the FITC signal detector, whereas PI binding was assessed by the Phycoerythrin emission signal detector).

### 2.12. Statistical Analysis

Statistical analyses were performed using Prism software (GraphPad Prism 8.2.1). A One-way ANOVA for data analysis was applied. *P* < 0.05 was considered statistically significant. All the experiments were done in triplicate, and the results are shown in means ± SD.

## 3. Results

### 3.1. Characterization

**Figure 1:**
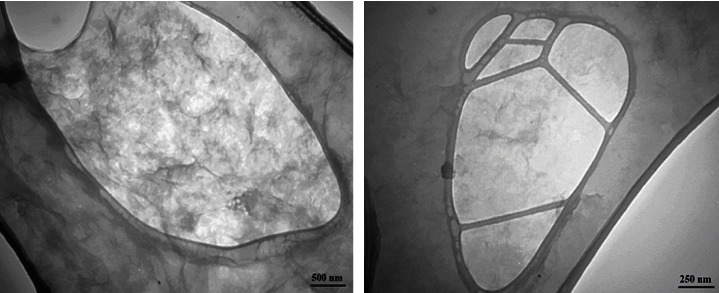
TEM results of GO-Alb-Cur-5FU-FA nanocomposite with different scale bars.

TEM analysis was used to study the structure and particle size. The morphology of the GO-Alb-Cur-5FU-FA nanocomposite is presented in Figure 1 which shows the presence of transparent sheets.

**Figure 2:**
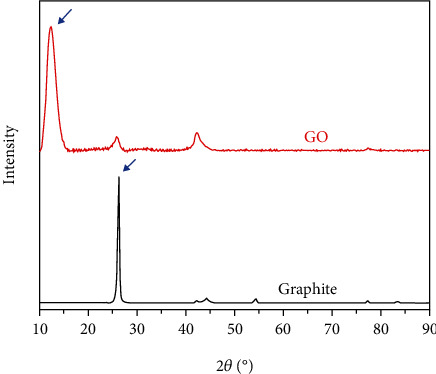
XRD analyze of GO and graphite.

The crystalline phase feature of graphite and GO was examined by X-ray diffraction (XRD) from 10 to 90 of 2*θ*, and the result is presented in Figure 2. Graphite shows an intense peak at 2*θ* of 26.62° corresponding to its 002-plane, and in the GO pattern, because of the intense oxidation, this peak disappeared, and a broad peak appeared at 2*θ* of 12.03° corresponding to 001-plane.

**Figure 3:**
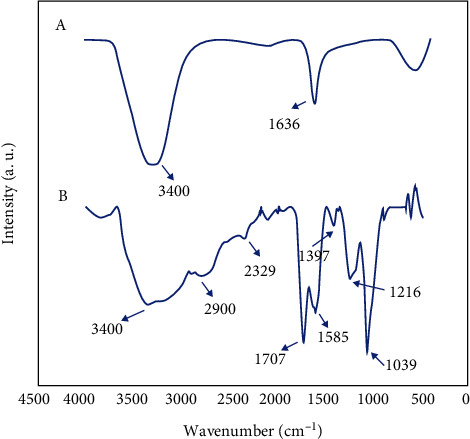
FTIR spectra of (a) GO and (b) GO-Alb-Cur-5FU.

To study the functional groups of the GO and GO-Alb-Cur-5FU, infrared analysis was used, and the results are given in Figure 3. In the GO case, the peaks at 1636 and 3400 cm^−1^ represent the C=C [34] and hydroxyl groups [35], respectively. The peak of 3400 cm^−1^ is outside the bell state, indicating the presence of both the hydroxyl groups belonging to the GO and albumin parts and the amine group belonging to the albumin structure [36]. The peak at 1707 cm^−1^ indicates the presence of the C=O functional group [37], and the peak at 2900 cm^−1^ is due to the C-H functional group [38] belonging to the albumin and folic acid parts of the nanocomposite. In addition, the peak observed at 1585 cm^−1^ is related to C═O stretching vibration in curcumin [39] (Figure 3).

### 3.2. Assessment of Loaded Drug

The amount of loaded drug was assessed using HPLC analysis. The results represented in Table 1 show the results of the amount of drugs loaded in nanoparticles for both Cur and 5FU drugs. The results show that the weight percentage for Cur and 5FU were 4.51 ± 0.42% and 6.89 ± 0.45% of the total weight of GO-Alb-Cur-5FU-FA nanoparticles, respectively.

### 3.3. Evaluation of FA Conjugation

HPLC analysis was used to determine the amount of conjugated FA by using the calculation of the FA weight (%) of total weight of nanoparticles. It showed that FA have 16.25 ± 1.04% of the total weight of GO-Alb-Cur-5FU-FA nanoparticles (Table 2).

### 3.4. Drug Release Study

**Figure 4:**
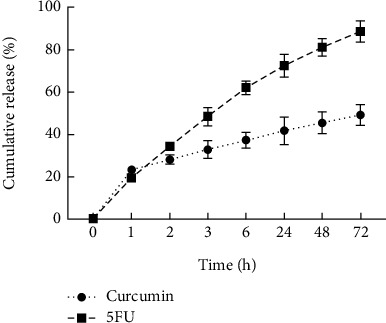
Cur and 5FU Release from GO-Alb-Cur-5FU-FA.

The results of the release evaluations are presented in Figure 4 and show that the release of the Cur was initiated at the first hour and reached 40% and 50% of the loaded Cur after 24 h and 72 h, respectively. These data confirm that about 70% of the loaded 5FU was released in the first 24 hours and 90% after 72 h.

### 3.5. Cytotoxicity Assessment

**Figure 5:**
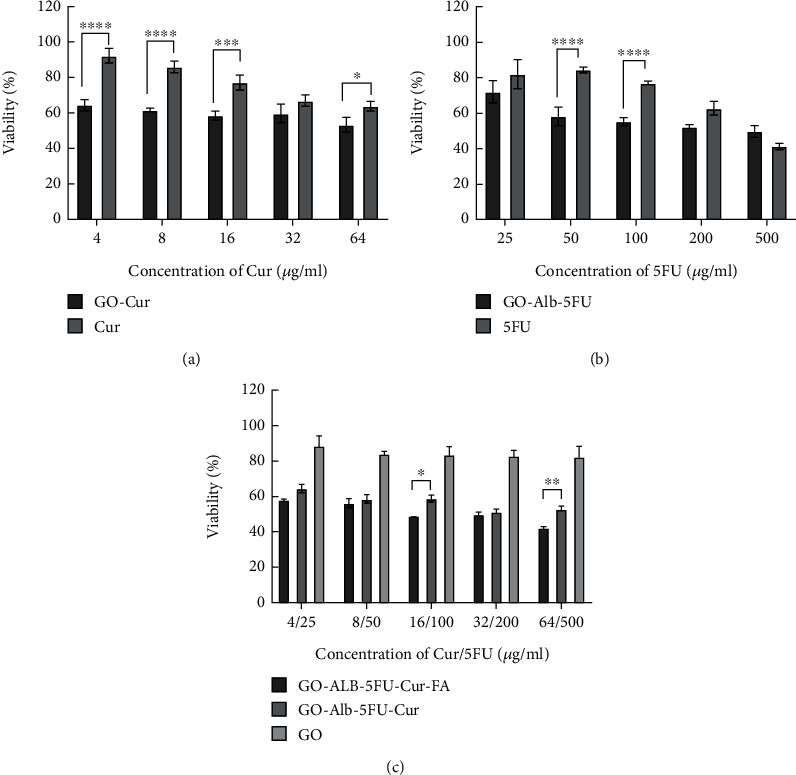
HT-29 cells viability treated by different groups of nanocomposites.

Cytotoxicity of GO, free 5FU, free Cur, GO-Cur, GO-Alb-5FU, GO-Alb-Cur-5FU, and GO-Alb-Cur-5FU-FA toward HT-29 and HUVEC cell lines was evaluated by MTT assay. The toxicity effect of free Cur and GO-Cur on HT-29 cells was studied, and the results are shown in Figure 5(a) which represent that loading Cur on graphene nanoparticles significantly increases its toxicity. The toxicity effect of free 5FU and GO-Alb-5FU on HT-29 cells was also investigated, and the results are given in Figure 5(b) which shows that loading 5FU on graphene nanoparticles in some concentrations of the drug significantly increases its toxicity effect.

The toxicity effects of GO-Alb-5FU-Cur-FA, GO-Alb-5FU-Cur, and GO on HT-29 cells were studied. The results showed that folic acid-modified nanoparticles containing both drugs significantly increased the toxicity effect compared to unmodified nanoparticles and graphene nanoparticles (Figure 5(c)).

**Figure 6:**
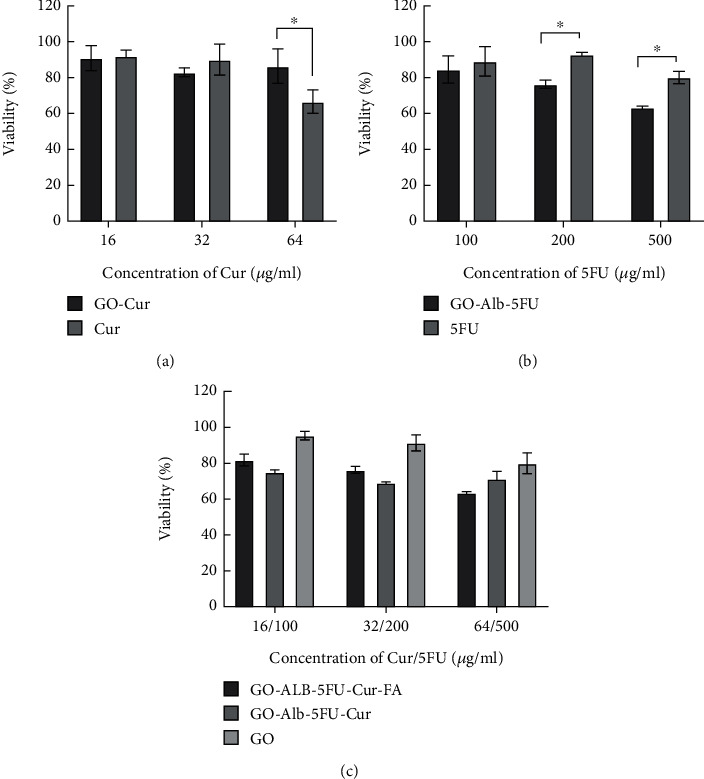
HUVEC cell viability treated by different groups of nanocomposites.

The toxicity of free Cur and GO-Cur on HUVEC cells was examined, and the results are given in Figure 6(a) which shows that Cur loaded on graphene nanoparticles significantly reduces its toxicity effect. The toxicity of free 5FU and GO-Alb-5FU on HUVEC cells was investigated, and the results are shown in Figure 6(b) which represents that GO-Alb-5FU has a significantly greater toxicity effect than free 5FU. The toxicity effect of GO, GO-Alb-5FU-Cur, and GO-Alb-5FU-Cur-FA on HUVEC cells was investigated. The results represented in Figure 6(c) showed that the nanoparticles modified with folic acid containing both drugs had no significant toxic effect compared to unmodified nanoparticles.

### 3.6. Apoptosis Evaluation

**Figure 7:**
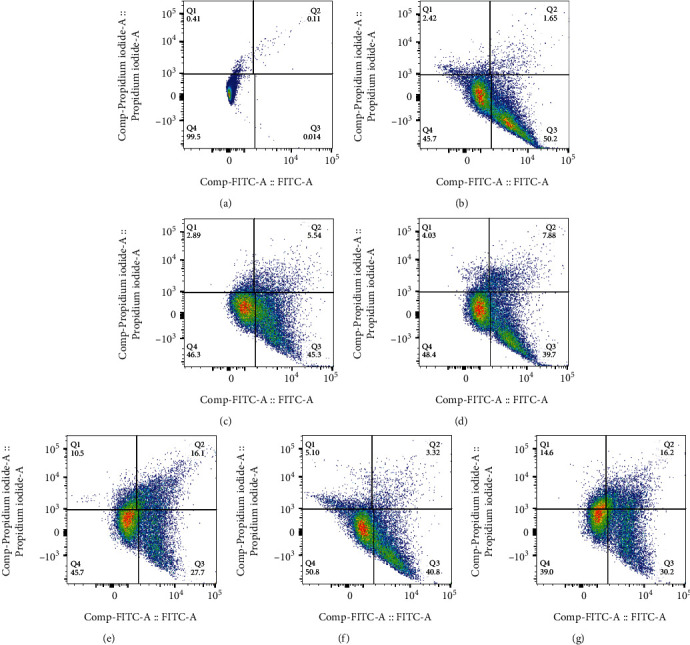
Flow cytometry results of (a) untreated HT-29 cell, (b) cell treated with 5FU, (c) GO-5FU, (d) Cur, (e) GO-Cur, (f) GO-Alb-Cur-5FU, and (g) GO-Alb-Cur–5FU-FA.

The apoptotic effect of the nanocarrier on HT-29 and HUVEC cells was investigated using Annexin V and PI apoptosis kit with flow cytometry analysis. There are many symptoms in the apoptotic pathway that can be biologically measured. In this study, changes in the cytoplasmic membrane were used as an apoptosis sign. The transfer of the polar heads of phosphatidylserine from the inner monolayer of the cytoplasmic membrane to its outer surface is indicative of the early stages of apoptosis. Anxin V (an anticoagulant) protein has a high synthetic tendency to bind to serine phospholipids. On the other hand, by increasing the permeability of cell membranes in the late stage of the process of apoptosis and necrosis, PI dye penetrates the cell and stains the cell DNA. The flow cytometry table includes 4 segments: Q1 (necrosis cell), Q2 (late apoptosis), Q3 (early apoptosis), and Q4 (viable cells).  Figure 7(a) shows the flow cytometry analysis of untreated HT-29 cells as a control which shows most cells remain alive.

Figures 7(b) and 7(c) show that the HT-29 cells treated with GO-5FU had more apoptotic effect (early apoptosis and late apoptosis: 5.54% and 50.20%, respectively) than the cells treated with free 5FU (1.65% and 45.30%, respectively).

Figures 7(d) and 7(e) show that HT-29 cells treated with GO-Cur had a more apoptotic effect (late apoptotic effect 16.1 %) than free Cur (late apoptotic effect 7.88 %). However, the cells treated with GO-Cur had a lower initial apoptotic effect (27.7%) than free Cur (39.7%).

Figures 7(f) and 7(g) shows a lower late apoptotic effect (late apoptosis: 3.32%) in the cells treated with GO-Alb-Cur-5FU than GO-Alb-Cur-5FU-FA (late apoptosis:16.20%), while cells treated with folate-modified nanodrugs (GO-Alb-Cur-5FU-FA) showed a lower early apoptotic effect (early apoptosis 30.2%) than the cells treated with GO-Alb-Cur-5FU (early apoptosis 40.8%).

**Figure 8:**
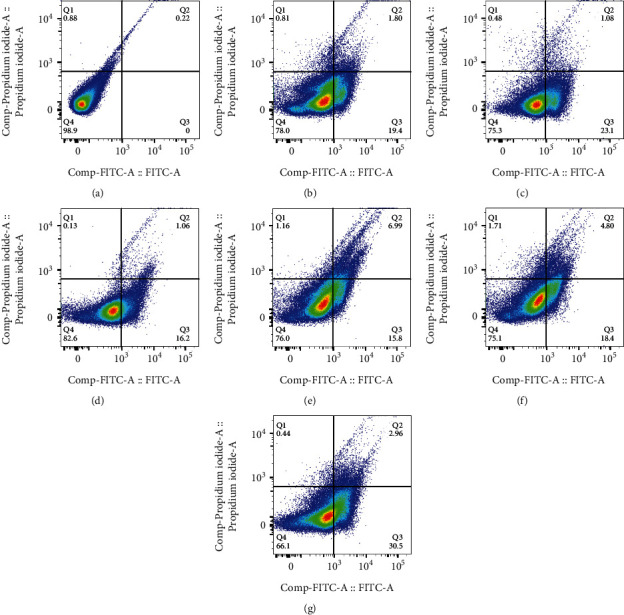
Flow cytometry results of (a) untreated HUVEC cell, (b) 5FU, (c) GO-5FU, (d) Cur, (e) GO-Cur, (f) GO-Alb-Cur-5FU, and (g) GO-Alb-Cur-5FU-FA.

Figure 8(a) shows the flow cytometry analysis results of the untreated HUVEC cells as a control, showing most cells remain alive. Figures 8(b) and 8(c) show that the HUVEC cells treated with GO-5FU had no significant effect in comparison with cells treated with free 5FU. However, cells treated with GO-5FU showed a higher early apoptotic effect (23.1%) than cells treated with free 5FU (19.4%). Figures 8(d) and 8(e) show a higher late apoptotic effect (6.99%) in HUVEC cells treated with GO-Cur than free Cur (1.06%). However, these treatments had not any significant differences in early apoptosis (15.80% and 16.20%).

Figures 8(f) and 8(g) show a higher late apoptotic effect (4.80%) in cells treated with the GO-Alb-Cur-5FU than in cells treated with GO-Alb-Cur-5FU-FA (2.96%), while the cells treated with GO-Alb-Cur-5FU-FA had a higher effect on early apoptosis (30.5%) than the cells treated with GO-Alb-Cur-5FU (18.4%).

## 4. Discussion

Cancer is one of the major health problems in the world, and although there are many treatments for cancer, the survival of cancer patients is still limited. Therefore, discovering new technologies that reduce damage to the body's natural cells and organs is a priority. 5FU is an anticancer drug that acts as an antimetabolic drug, exerts a cytotoxic effect, and inhibits DNA production. Today, in addition to chemical medicines, herbal medicines such as Cur are used to treat cancer. Cur has effects such as anti-inflammatory, antioxidant, and anticoagulant activities. Cur is a hydrophobic drug that is usually used in nanocarriers such as nanoliposomes to increase its solubility, absorption, and efficacy [9, 17, 25, 40]. In recent years, several efforts have been made to develop GO-based nanocarriers, which have been widely used in gene and drug delivery. Since folate receptors are highly expressed in the membranes of many cancer cells, such as colorectal cells, folic acid has also been used to target these cells [29]. In this study, the prepared graphene oxide was modified with folic acid to target human colorectal cancer (HT29) cell lines.

The toxicity studies conducted here showed that loading Cur or 5FU into graphene nanoparticles significantly increases their anticancer efficiency. These results are consistent with the study done by Al-Ani et al. which evaluated the effect of graphene oxide and the hybrid Cur nanocomposite on colon cancer cells. This nanocomposite inhibited colon cancer cells depending on the dose and time of use [41].

Yang et al. studied the synergistic effect of 5FU and Cur on the MCF7 cancer line and showed that the combination of these two drugs increases cytotoxicity and decreases IC50 concentration [42]. In another study in 2021 by Yang et al., the synergistic effect of 5FU and Cur with folic acid on the MCF7 cancer line was evaluated, and the results showed that the combination of the two drugs increased the toxicity and lowered the IC50 concentration [34]. The results of cytotoxicity assessment in the current study show that the IC50 combination of 5FU and Cur on HT-29 cells is approximately 200 *μ*g mL^−1^. Also, the modification of the nanoparticles with folic acid increased the toxicity effect compared to unmodified nanoparticles. These differences in cytotoxicity can be due to the folic acid, different surface loads, and the presence of FR in HT-29 cells. In 2010, in a study done by Lu et al., molecular graphene oxide and folic acid (FA) were used to load doxorubicin as an anticancer drug. Their results showed that the combined drugs with graphene oxide and folic acid were more effective than a single drug. The reported higher efficiency can be due to folic acid receptors on MCF-7 cells [43].

Based on the results reported above on HUVEC cells, the loading of 5FU into graphene nanoparticles significantly increased its toxicity effect compared to free. Mirzaghavami et al. in 2021 investigated the effects of 5FU on HUVEC cells and showed that increasing the concentration of 5FU inhibits thymine synthesis in DNA and causes the destruction of HUVEC cells, which is consistent with the results of this study [44].

Flow cytometry results show that Cur and 5FU have a more lethal effect in both cell lines when loaded into GO and also bounded to folic acid than in free forms. In the study of Zhou et al., the hybrid effect of Cur and 5FU on a gastric cancer cell line was examined, and the results showed that the combined effect of these two drugs causes a higher rate of apoptosis in the BGC-823 cells compared to Cur alone [45].

## 5. Conclusion

In this study, we prepared a folic acid-functionalized albumin-coated GO nanocomposite containing 5FU and Cur to evaluate its effect on human colon cancerous cells (HT-29). The nanocomposite was characterized by different analyses, and their cytotoxicity was evaluated by MTT and flow-cytometric apoptosis assays. The results demonstrated that folic acid-modified graphene oxide nanoparticles containing Cur and 5FU have delivered drugs to HT-29 cells in comparison with normal cells and may have a high capability to deliver drugs to HT-29 cells. Therefore, the surface modification of graphene oxide nanoparticles with folic acid could increase its capability and selectivity for active targeted delivery. Moreover, the findings showed that the designed delivery system can be utilized as a promising solution for active, targeted drug delivery.

## Figures and Tables

**Table 1:** Assessment of drugs loaded in nanoparticles in the studied graphene nanoparticles.

	Sample	The amount of Cur loaded in nanoparticles (%)	The amount of 5FU loaded in nanoparticles (%)
1	GO-Cur	11.1 ± 0.5	0
2	GO-Cur-FA	7.81 ± 0.45	0
3	GO-Alb--FA	0	0
4	GO-Alb-5FU	0	7.08 ± 0.42
5	GO-Alb-Cur-5FU	5.31 ± 0.46	9.57 ± 0.68
6	GO-Alb-5FU-FA	0	5.41 ± 0.25
7	GO-Alb-Cur-5FU-FA	4.51 ± 0.42	6.89 ± 0.45

**Table 2:** Evaluation of FA conjugated in the studied graphene nanoparticles.

	Sample	FA weight (%) of total weight of nanoparticles
1	GO-Alb-FA	18.16 ± 1.05
2	GO-Alb-5FU	0
3	GO-Alb-5FU-FA	17.05 ± 1.16
4	GO-Cur	0
5	GO-Cur-FA	28.47 ± 2.25
6	GO-Alb-Cur-5FU	0
7	GO-Alb-Cur-5FU-FA	16.25 ± 1.04

## Data Availability

We prefer that research information be emailed to other researchers upon their request.

## References

[1] Hassanpour S. H., Dehghani M. (2017). Review of cancer from perspective of molecular. *Journal of Cancer Research and Practice*.

[2] Dahan L., Sadok A., Formento J. L., Seitz J. F., Kovacic H. (2009). Modulation of cellular redox state underlies antagonism between oxaliplatin and cetuximab in human colorectal cancer cell lines. *British Journal of Pharmacology*.

[3] Montminy E. M., Zhou M., Maniscalco L. (2021). Contributions of adenocarcinoma and carcinoid tumors to early-onset colorectal cancer incidence rates in the United States. *Annals of Internal Medicine*.

[4] Zhou J., Zheng R., Zhang S. (2021). Colorectal cancer burden and trends: comparison between China and major burden countries in the world. *Chinese Journal of Cancer Research*.

[5] Buskaran K., Hussein M. Z., Moklas M. A. M., Masarudin M. J., Fakurazi S. (2021). Graphene oxide loaded with protocatechuic acid and chlorogenic acid dual drug nanodelivery system for human hepatocellular carcinoma therapeutic application. *International Journal of Molecular Sciences*.

[6] Vecchia S., Sebastián C. (2020). Metabolic pathways regulating colorectal cancer initiation and progression. *Seminars in Cell & Developmental Biology*.

[7] Tacconi C., Ungaro F., Correale C. (2019). Activation of the VEGFC/VEGFR3 pathway induces tumor immune escape in colorectal cancer. *Cancer Research*.

[8] Mahmoudi R., Ashraf Mirahmadi-Babaheidri S., Delaviz H. (2021). RGD peptide-mediated liposomal curcumin targeted delivery to breast cancer cells. *Journal of Biomaterials Applications*.

[9] Mahmoudi R., Hassandokht F., Ardakani M. T. (2021). Intercalation of curcumin into liposomal chemotherapeutic agent augments apoptosis in breast cancer cells. *Journal of Biomaterials Applications*.

[10] Li J., Hou N., Faried A., Tsutsumi S., Takeuchi T., Kuwano H. (2009). Inhibition of autophagy by 3-MA enhances the effect of 5-FU-induced apoptosis in colon cancer cells. *Annals of Surgical Oncology*.

[11] Basnet P., Skalko-Basnet N. (2011). Curcumin: an anti-inflammatory molecule from a curry spice on the path to cancer treatment. *Molecules*.

[12] Mahmoudi R., Honarmand Z., Karbalay-Doust S., Jafari-Barmak M., Nikseresht M., Noorafshan A. (2017). Using curcumin to prevent structural impairments of testicles in rats induced by sodium metabisulfite. *EXCLI Journal*.

[13] Debele T. A., Peng S., Tsai H.-C. (2015). Drug carrier for photodynamic cancer therapy. *International journal of Molecular Sciences*.

[14] Sadegh N., Haddadi H., Arabkhani P., Asfaram A., Sadegh F. (2021). Simultaneous elimination of rhodamine B and malachite green dyes from the aqueous sample with magnetic reduced graphene oxide nanocomposite: optimization using experimental design. *Journal of Molecular Liquids*.

[15] Sawant V., Tawade B., Desai V., Dongare B., Nipane S. (2022). Graphene-tethered 5-fluorouracil-loaded ZnO nanocomposites for pH-responsive enhanced efficacy in drug delivery on MCF-7 cells. *Biomaterials*.

[16] Hashemi S.-S., Rajabi S.-S., Mahmoudi R., Ghanbari A., Zibara K., Barmak M. J. (2020). Polyurethane/chitosan/hyaluronic acid scaffolds: providing an optimum environment for fibroblast growth. *Journal of Wound Care*.

[17] Hashemi S.-S., Saadatjo Z., Mahmoudi R. (2022). Preparation and evaluation of polycaprolactone/chitosan/Jaft biocompatible nanofibers as a burn wound dressing. *Burns*.

[18] Bardania H., Mansouri R., Tahoori M. T. (2021). Switch off inflammation in spleen cells with CD40-targeted PLGA nanoparticles containing dimethyl fumarate. *Colloids and Surfaces B: Biointerfaces*.

[19] Doustimotlagh A. H., Kokhdan E. P., Vakilpour H. (2020). Protective effect of Nasturtium officinale R. Br and quercetin against cyclophosphamide-induced hepatotoxicity in rats. *Molecular Biology Reports*.

[20] Hiremath C. G., Kariduraganavar M. Y., Hiremath M. B. (2018). Synergistic delivery of 5-fluorouracil and curcumin using human serum albumin-coated iron oxide nanoparticles by folic acid targeting. *Progress in Biomaterials*.

[21] Loutfy S. A., Salaheldin T. A., Ramadan M. A., Farroh K. Y., Abdallah Z. F., Youssef T. (2017). Synthesis, characterization and cytotoxic evaluation of graphene oxide nanosheets: in vitro liver cancer model. *Asian Pacific journal of cancer prevention: APJCP*.

[22] Wang K., Ruan J., Song H. (2010). Biocompatibility of graphene oxide. *Nanoscale Research Letters*.

[23] Sharma H., Mondal S. (2020). Functionalized graphene oxide for chemotherapeutic drug delivery and cancer treatment: a promising material in nanomedicine. *International Journal of Molecular Sciences*.

[24] Gupta N., Aggarwal N. (2007). Stomach-specific drug delivery of 5-fluorouracil using floating alginate beads. *AAPS PharmSciTech*.

[25] Salehi Najafabadi P., Delaviz H., Asfaram A. (2022). Evaluation of the biodistribution of arginine, glycine, aspartic acid peptide-modified Nanoliposomes containing curcumin in rats. *Iranian Journal of Biotechnology*.

[26] Ghaffari S.-B., Sarrafzadeh M.-H., Fakhroueian Z., Shahriari S., Khorramizadeh M. R. (2017). Functionalization of ZnO nanoparticles by 3-mercaptopropionic acid for aqueous curcumin delivery: synthesis, characterization, and anticancer assessment. *Materials Science and Engineering: C*.

[27] Ghaffari S.-B., Sarrafzadeh M.-H., Salami M., Khorramizadeh M. R. (2020). A pH-sensitive delivery system based on N-succinyl chitosan-ZnO nanoparticles for improving antibacterial and anticancer activities of curcumin. *International Journal of Biological Macromolecules*.

[28] Yang R., An Y., Miao F., Li M., Liu P., Tang Q. (2014). Preparation of folic acid-conjugated, doxorubicin-loaded, magnetic bovine serum albumin nanospheres and their antitumor effects in vitro and in vivo. *International Journal of Nanomedicine*.

[29] Huang P., Xu C., Lin J. (2011). Folic acid-conjugated graphene oxide loaded with photosensitizers for targeting photodynamic therapy. *Theranostics*.

[30] Ghaffari S.-B., Sarrafzadeh M.-H., Fakhroueian Z., Khorramizadeh M. R. (2019). Flower-like curcumin-loaded folic acid-conjugated ZnO-MPA- *β*cyclodextrin nanostructures enhanced anticancer activity and cellular uptake of curcumin in breast cancer cells. *Materials Science and Engineering: C*.

[31] Handali S., Moghimipour E., Kouchak M. (2019). New folate receptor targeted nano liposomes for delivery of 5-fluorouracil to cancer cells: strong implication for enhanced potency and safety. *Life Sciences*.

[32] Smina C., Lalitha P., Sharma S., Nagabhushana H. (2021). Screening of anti-cancer activity of reduced graphene oxide biogenically synthesized against human breast cancer MCF-7 cell lines. *Applied Nanoscience*.

[33] Baneshi M., Dadfarnia S., Haji Shabani A. M., Sabbagh S. K., Bardania H. (2022). AS1411 aptamer-functionalized graphene oxide-based nano-carrier for active-target and pH-sensitive delivery of curcumin. *Journal of the Iranian Chemical Society*.

[34] Umamaheswari S., Murali M. (2013). FTIR spectroscopic study of fungal degradation of poly (ethylene terephthalate) and polystyrene foam. *Chemical Engineer*.

[35] WOS R., Sabiqoh Z., Ratnaningsih E., Hertadi R. (2021). Isolation and Characterization of Poly-(R)-3-hydroxybutyrate Produced by Bacillus thuringiensis TH-01. *Key Engineering Materials*.

[36] Pang Y., Chen Z., Zhao R. (2021). Facile synthesis of easily separated and reusable silver nanoparticles/aminated alkaline lignin composite and its catalytic ability. *Journal of Colloid and Interface Science*.

[37] Song S.-W., Hidajat K., Kawi S. (2005). Functionalized SBA-15 materials as carriers for controlled drug delivery: influence of surface properties on matrix− drug interactions. *Langmuir*.

[38] Dong H., Virtanen S. (2022). Influence of bovine serum albumin on biodegradation behavior of pure Zn. *Journal of Biomedical Materials Research Part B: Applied Biomaterials*.

[39] Su Y., Chen Y., Zhang L. (2022). Synthesis and characterization of lotus seed protein-based curcumin microcapsules with enhanced solubility, stability, and sustained release. *Journal of the Science of Food and Agriculture*.

[40] Moslemi Z., Bardania H., Gheitasi I. (2021). Liposome extract of Stachys pilifera Benth effectively improved liver damage due to bile duct ligation rats. *Oxidative Medicine and Cellular Longevity*.

[41] Al-Ani L. A., Yehye W. A., Kadir F. A. (2019). Hybrid nanocomposite curcumin-capped gold nanoparticle-reduced graphene oxide: anti-oxidant potency and selective cancer cytotoxicity. *PLoS One*.

[42] Yang Y., Wang S., Wang C., Tian C., Shen Y., Zhu M. (2019). Engineered targeted hyaluronic acid–glutathione-stabilized gold nanoclusters/graphene oxide–5-fluorouracil as a smart theranostic platform for stimulus-controlled fluorescence imaging-assisted synergetic chemo/phototherapy. *Chemistry–An Asian Journal*.

[43] Lu T., Nong Z., Wei L. (2020). Preparation and anti-cancer activity of transferrin/folic acid double-targeted graphene oxide drug delivery system. *Journal of Biomaterials Applications*.

[44] Mirzaghavami P. S., Khoei S., Khoee S., Shirvalilou S., Mahdavi S. R., Mahabadi V. P. (2021). Radio-sensitivity enhancement in HT29 cells through magnetic hyperthermia in combination with targeted nano-carrier of 5-Flourouracil. *Materials Science and Engineering: C*.

[45] Zhou X., Wang W., Li P. (2016). Curcumin enhances the effects of 5-fluorouracil and oxaliplatin in inducing gastric cancer cell apoptosis both in vitro and in vivo. *Oncology Research*.

